# Looking backward in time to define the chronology of metastasis

**DOI:** 10.1038/s41467-020-16995-y

**Published:** 2020-06-25

**Authors:** Zheng Hu, Christina Curtis

**Affiliations:** 1 Department of Medicine, Division of Oncology, Stanford University School of Medicine, Stanford, CA USA; 2 Department of Genetics, Stanford University School of Medicine, Stanford, CA USA; 3 Stanford Cancer Institute, Stanford University School of Medicine, Stanford, CA USA

**Keywords:** Cancer epigenetics, Cancer genomics

## Abstract

The timing of cancer metastasis has implications for treatment and prevention. Traditional forward-time views of metastasis assume it occurs late during evolution. However, looking backward in time reveals metastasis often occurs prior to clinical detection of primary tumors.

## Forward-time models of metastatic seeding

Systemic spread of a primary tumor to distant organ sites is the primary cause of cancer mortality. Effective treatment and prevention of metastasis remains a major objective of oncology and should be informed by an understanding of the natural history of this process. In particular, it is of interest to know when cells disseminate from a malignant tumor and seed distant metastases. Due to the limitations of current imaging techniques, many cancers are only detected when they have reached a relatively large size (e.g. >1 cm^3^). As such, when metastatic seeding initiates and the time required for growth from a micro-metastatic lesion to an overt metastasis is poorly understood^1^. Recently, insights into the timing and molecular determinants of this lethal and occult process in the human system has been enabled by genomic profiling of paired primary tumors and metastases and the development of new computational approaches.

### Linear progression—minor clone model

**Fig. 1:**
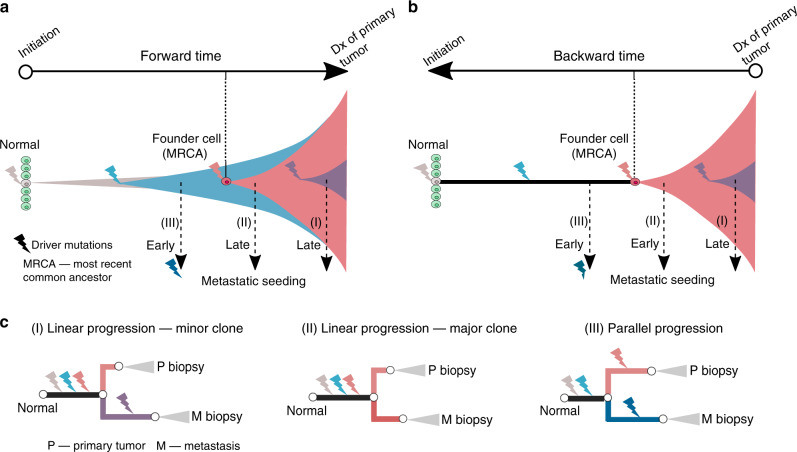
Models and chronology of metastatic dissemination. **a** Forward-time models and the chronology of metastatic dissemination. Metastasis occurs late in linear progression (either(I)—minor clone model or (II)—major clone model) and early in the parallel progression model (III). **b** Backward-time models and the chronology of metastatic dissemination. Metastasis commonly occurs several years prior to detection of the primary tumor, consistent with early dissemination from the major or dominant clone after emergence of the cancerous founder cell. **c** Driver gene heterogeneity in bulk sequencing of paired primary tumors and metastases under different models. In the minor clone model (I) of linear progression, metastases typically harbor “private” driver mutations which are present at low frequency in the primary tumor and undetectable in bulk sequencing. In contrast, the origin of metastasis from a major clone (II) typically leads to driver gene homogeneity. Parallel progression (III) characteristically leads to high driver gene heterogeneity where both the primary tumor and metastasis acquire private driver mutations after dissemination. Dx, diagnosis.

The forward-time view of the emergence of metastatic phenotypes (e.g. invasion, dissemination and colonization) posits that metastasis is a late event relative to tumor initiation (the occurrence of the first cancer-initiating mutation). This has formed the basis for the linear progression model^1^. For instance, it has been estimated that two to eight driver mutations and typically more than 10 years are required to convert a normal cell to a cancerous state that outcompetes its precancerous counterparts to form a malignant tumor (Fig. 1a)^2,3^. Based on mutation data in colorectal cancers, Jones et al.^2^ estimated that ~20 years are required for a benign lesion to evolve into an advanced metastatic cancer. Yachida et al.^3^ similarly estimated that pancreatic cancer acquires metastatic ability more than 10 years after the occurrence of the first cancer-initiating mutation. Metastasis is thus commonly described as a late event in the molecular history of cancer relative to occurrence of the cancer-initiating mutation.

The classical linear progression model posits that only a small subpopulation (or subclone) in the primary tumor evolves to acquire specialized genetic alterations that bestow metastatic competence^1^, corresponding to scenario (I) in Fig. 1a, b. In other words, the seeds of metastasis derive from a minor mutant subpopulation and emerge late in the evolution of the primary tumor. The minor-clone hypothesis is favored by clinical and experimental observations indicating that metastatic seeding is inefficient where an estimated <0.02% of disseminated tumor cells (DTCs) successfully seed metastases^4^.

### Linear progression—major clone model

Genomic data from paired primary tumors and metastases are at odds with the minor clone hypothesis. In particular, if metastasis is seeded by a minor subclone that has acquired additional mutations, these alterations are unlikely to be detected by bulk sequencing of the primary tumor and will appear “metastasis-specific” in the sequencing data (Fig. 1c, scenario (I)). The detection of such low frequency mutations is further aggravated by the fact that typically a small fraction of total tumor volume is biopsied and subject to sequencing. Consequently, if the minor clone model dominates, metastases should possess private driver mutations that are not detected in the paired primary tumor (Fig. 1c, scenario (I)). Instead, genomic data across a variety of cancer types have documented high driver gene concordance between primary tumors and metastases with few metastasis-private driver mutations identified^1,5,6^. For instance, in our recent study^6^, we analyzed exome sequencing data from 457 paired primary tumor and metastatic samples from 136 patients with colorectal, lung, or breast cancer and found that 84, 86 and 59% of driver mutations were shared in primary tumor and metastases in these common cancer types, respectively. Of note, the lower prevalence of shared driver mutations in breast cancer is associated with adjuvant treatment which likely selects for drug resistant mutations in a minor clone in the primary tumor, leading to the higher prevalence of metastasis-specific driver mutations in treated metastases. In this regard, metastasis-private mutations are not drivers of cancer spread but instead are associated with drug resistance. Taken together, the low genomic divergence between primary tumors and metastases across diverse epithelial tumors suggests that metastases commonly derive from a major clone (Fig. 1a–c, scenario (II)) in the primary tumor.

The origin of metastasis from a major mutant clone in the primary tumor is in line with the clonal dominance hypothesis proposed by Kerbel et al.^7^. This model assumes that once a metastatic subclone emerges within a primary tumor, cells from this subclone with high fitness will outcompete and dominate the tumor mass itself, resulting in genetic and phenotypic similarities between the primary tumor and metastasis. This hypothesis was originally derived from xenograft experiments using the integration of foreign DNA as a means to genetically tag diverse tumor cell populations^7^. These studies suggest that the same cell lineage dominates both the primary site and metastasis consistent with a competitive growth advantage for the metastatic lineage in the primary tumor. A recent study by Chen et al.^8^ employed a CRISPR/Cas9 screen to identify metastatic drivers in mouse models. These data indicate that very few metastasis-competent lineages survive and ultimately dominate both the primary tumor and metastatic sites. The clonal dominance model is also consistent with early observations that gene expression profiling of primary tumors can predict distant recurrence^9^.

### Parallel progression

The parallel progression model assumes that metastatic seeding occurs early during tumorigenesis, typically before emergence of the cancerous founder cell (or the most recent common ancestor, MRCA) of the primary tumor^10^. After dissemination, both the primary tumor and metastasis independently evolve and acquire additional driver mutations, leading to genomic divergence (Fig. 1a–c – scenario (III)). In summary, parallel progression is characterized by early metastatic spread, relatively short truncal branches and the occurrence of private driver mutations in both primary and metastatic lineages. While this pattern has been observed in some studies (reviewed in ref. ^10^), primary tumor-private and metastasis-private driver mutations are rarely observed in bulk genomics data in the same patient^1,6^.

## Backward-time models of metastatic seeding

The time from emergence of a cancerous founder cell to clinical detection of the primary tumor has been estimated to be on the order of a few years, typically less than ten^2,3,6^.

Although this window is much shorter than pre-malignant evolution which can occur over decades (Fig. 1a), metastasis can still occur early relative to clinical detection. For instance, a metastasis might be seeded several years prior to diagnosis of primary. In other words, metastasis can occur late relative to tumor initiation, but early relative to clinical detection (Fig. 1b).

Early studies (reviewed in ref. ^11^) analyzed tumor doubling times across human cancers and metastases and extrapolated back using a mathematical model of tumor growth to estimate metastatic timing. These estimates suggest that metastasis occurs years before diagnosis of the primary tumor. Hanin et al.^12^ developed a stochastic mathematical model of the sizes of multiple metastases in an individual patient, and estimated that metastasis in breast cancer can occur early when the primary tumor is <1 mm in diameter. Intriguingly, these results suggest a long latency between dissemination and metastatic regrowth^12^. Haeno et al.^13^ used a similar mathematical framework to model metastatic burden estimates based on imaging from the time of diagnosis to death in pancreatic cancers obtained via rapid autopsy and reasoned that all primary cancers are likely to harbor metastatic clones at diagnosis.

Recent studies have performed phylogenetic analysis of genomic data to calibrate the timing of metastatic divergence, yielding genomic support for clinically early dissemination. For instance, Zhao et al.^14^ reconstructed the phylogenies of primary tumors and matched metastases (*n* = 2–7 per patient) in 40 patients with 13 cancer types and found that in nearly 90% of patients, genetic divergence of the first metastatic lineage had occurred by the time of diagnosis of the primary tumor while some lineages diverged years earlier. Noorani et al.^15^ performed phylogenetic analysis of paired primary and metastatic samples in a cohort of esophageal adenocarcinomas and showed that in 90% of patients, multiple subclones spread from the primary site to form multiple metastases. Interestingly, the metastatic lineages exhibited a depletion of clock-like mutational signature (predominantly C > T transitions in NpCpG trinucleotide context), consistent with rapid spread after emergence of the malignant founder clone.

It should be noted, however, that the chronological time of metastatic dissemination cannot readily be resolved by phylogenetic inference since the divergence time on the phylogenetic tree is not equivalent to the time of dissemination when metastasis founder cells leave the primary tumor and seed the metastases^5^. Additionally, phylogenetic approaches do not account for potential biases caused by limited tissue sampling, spatial structure within solid tumors, clonal selection, and mutation rate variation etc. To address these issues, we developed a spatial computational model of tumor growth and metastasis and leveraged a statistical inference framework to quantify the size (age) of the primary tumor at the time of metastatic dissemination^5^. Applying this method to a cohort of paired primary colorectal cancers and metastases revealed early metastatic seeding when the primary tumor was small and clinically undetectable (typically <0.1 cm^3^). In a follow-up study, we analyzed genomic data from colorectal, breast and lung cancers and estimated that metastatic dissemination commonly occurs 2–4 years prior to diagnosis of the primary tumor^6^. Of note, adjuvant treatment was associated with the presence of private driver mutations in metastases and later metastatic seeding, suggesting that treatment selects for drug-resistant metastatic clones and can delay metastatic outgrowth. Taken together, accumulating lines of evidence suggest that metastatic spread can occur rapidly following the emergence of invasive malignant cells, often several years prior to the diagnosis of the primary tumor. In colorectal cancer candidate biomarkers associated with early metastasis have been identified^5^ and it will be important to further validate these findings as well as to define such associations in other tumor types. Additionally, it will be crucial to understand factors that prevent metastatic outgrowth, including the contribution of immune surveillance.

## Conclusions

The traditional forward-time view of metastasis has favored the assumption that systemic spread is a late event in a tumor’s lifespan. This is certainly the case if the clock is assumed to start during tumor initiation. Indeed, it is appreciated that tumorigenesis occurs over many years (potentially decades) through a multi-step process where driver mutations can precede diagnosis of the primary tumor by years. At the same time, inference of metastatic timing from cancer genomic data suggests that this can occur years prior to clinical detection. Genomic data further suggest that in many cancers metastases are commonly seeded by a major mutant clone in the primary tumor. These observations thus help to resolve the controversy regarding early versus late metastatic seeding and the attendant clinical implications.
